# Evaluating diagnostic and management agreement between audiology and ENT: a prospective inter-rater agreement study in a paediatric primary contact clinic

**DOI:** 10.1186/s12887-022-03695-3

**Published:** 2022-11-08

**Authors:** Jennifer Eakin, Simone Michael, Christopher Payten, Tamsin Smith, Vicky Stewart, Elle Noonan, Kelly A. Weir

**Affiliations:** 1 Department of Speech Pathology and Audiology, Gold Coast Hospital and Health Service, 1 Hospital Boulevard, Gold Coast, Southport, QLD 4215 Australia; 2 Department of Physiotherapy, Gold Coast Hospital and Health Service, Gold Coast, QLD Australia; 3 Head, Neck, Oral and Neurosurgical Services, Gold Coast Hospital and Health Service, Gold Coast, QLD Australia; 4 Department of Allied Health Research, Gold Coast Hospital and Health Service, Gold Coast, QLD Australia; 5 Menzies Health Institute Queensland, Griffith University, Gold Coast, QLD Australia

**Keywords:** Audiology, Otolaryngology, Allied health primary contact model of care, Inter-rater agreement, Otitis media, Hearing loss

## Abstract

**Background:**

Ear, Nose and Throat (ENT) primary contact models of care use audiologists as the first triage point for children referred to ENT for middle ear and hearing concerns; and have shown reduced waiting time, improved ENT surgical conversion rates and increased service capacity. This study aimed to investigate ‘safety and quality’ of the model by looking at agreement between audiologists’ and an ENT’s clinical decisions.

**Methods:**

We performed an inter-rater agreement study on diagnosis and management decisions made by audiologists and an ENT for 50 children seen in an Australian hospital’s ENT primary contact service, and examined the nature and patterns of disagreements.

**Results:**

Professionals agreed on at least one site-of-lesion diagnosis for all children (100%) and on the primary management for 74% (Gwet’s AC1 = 0.67). Management disagreements clustered around i) providing ‘watchful waiting’ versus sooner medical opinion (18%), and ii) providing monitoring versus discharge for children with no current symptoms (8%). There were no cases where the audiologist recommended discharge when the ENT recommended further medical opinion.

**Conclusions:**

Our novel research provides further evidence that Audiologist-led primary contact models for children with middle ear and hearing concerns are safe as well as efficient.

**Supplementary Information:**

The online version contains supplementary material available at 10.1186/s12887-022-03695-3.

## Background

Patients with non-urgent Ear, Nose and Throat (ENT) concerns are waiting longer than clinically recommended to access specialist opinion within the Australian public health system. At Gold Coast University Hospital (GCUH, an Australian public hospital), more than half of patients triaged by an ENT specialist as requiring care within three months or one year are exceeding these timeframes due to lengthy waiting lists [1]. Children referred for hearing concerns and middle ear conditions such as otitis media are within this population, waiting longer than appropriate due to lower clinical prioritisation [2]. Chronic, untreated middle ear disease and associated conductive hearing loss in children has the potential to negatively impact speech and language development, auditory processing skills and academic achievement [3, 4] and, thus presents a challenge for public health systems to improve access to timely medical intervention.

Allied health primary contact models of care, which focus on optimising or extending the scope of allied health practitioners (AHPs), have been successfully implemented to address the issue of lengthy ENT waiting lists [5–8]. Under these models, patients referred by their general practitioner (GP) to public ENT services for specialist opinion and identified through referral triage by the ENT specialist for suitability are instead seen by an AHP for conditions related to their expertise, before or in lieu of the ENT specialist. Audiology-led primary contact clinics for children referred for middle ear and hearing concerns have been increasingly implemented in Australia with a range of positive outcomes including timelier access to initial diagnostic appointments, and to medical and surgical treatment [5, 9]. Audiologists can independently manage and discharge between 32–42% of children seen in the audiology-led primary contact clinics through education, reassurance or onward referral for speech and language intervention; and the surgical conversion rate for children seeing the ENT specialist is increased, optimising the function of ENT specialist clinics [5, 10, 11]. These clinics are estimated to increase the capacity of paediatric ENT specialist outpatient services by 77% [12].

In 2016, Gold Coast Health introduced an audiology-led paediatric primary contact service (ALP-PCS) to help manage the local demand for ENT specialist care. This service reduced the initial appointment wait time for children with middle ear and hearing concerns from 240 to 83 days, with 32% of children discharged back to their GP without the need for an ENT review [5].

## Objectives

While ALP-PCS are now recognised as a successful strategy for improving access to care, to date, no literature has reported on the diagnostic and management accuracy of these alternative pathways as the primary research outcome. This exploratory study aimed to investigate agreement between audiologists and an ENT specialist on site-of-lesion diagnosis and management plans for children referred into the ALP-PCS for middle ear or hearing concerns. Where disagreement occurred, the nature and patterns of that disagreement were examined to better understand the impact on clinical outcomes and patient safety. Whilst audiologists and ENT specialists bring a different perspective and expertise to the child’s initial appointment, in Australia, both professionals utilise the results of objective audiological testing such as air and bone conduction hearing thresholds and middle ear pressure and compliance readings (typically performed by the audiologist and available to the ENT at time of the medical review) to help form a diagnosis and guide decision making along best practice management models for middle ear and hearing concerns. Therefore, it is hypothesised that agreement between the two professionals on these metrics will be substantial.

## Materials and methods

### Ethical approval and consent to participate

Ethics approval was gained from the Gold Coast Hospital and Health Service Human Research and Ethics committee (HREC/17/QGC/121). All parents/legal guardians provided written and informed consent.

### Study design and site

This prospective cohort inter-rater agreement study was undertaken at the GCUH with reference to the ‘Guidelines for Reporting Reliability and Agreement Studies (GRRAS)’[13].

### Sample size

This sample size was determined on the advice of a biostatistician. We hypothesized that there should be at least substantial agreement between the audiologists and ENT specialist. The minimum value expected for Cohen’s Kappa was therefore set at 0.7, that is, the middle of the “substantial” agreement category on the Landis and Koch (1977) scale [14] and sample size set to detect a true level of agreement of 0.9 (“almost perfect”). The sample size was then calculated to ensure an estimated kappa would be accurate to within the bounds of one of the higher Landis and Koch categories. A sample size estimation of 49 was calculated based on comparisons of 4 to 10 categories for a power of 80% and type 1 error of 0.05 using the published tables of Bujang and Baharum (2017) [15].

### Participants

There were 110 children who met the study inclusion criteria and attended the ALP-PCS during the data collection period 11/7/2018 to 1/2/2019. Children were allocated appointments by the hospital referral team based on referral date and parent preference. Only children seen when the principal investigator was present in clinic were considered for the study (*N* = 51). One parent did not consent to the study, leaving a sample of 50 children. A retrospective chart review of the 50 children who were not considered for the study showed similar referral characteristics, with the most common reasons for referral in both groups being recurrent otitis media followed by hearing concerns, then speech delay.

#### Study Inclusion Criteria

**Fig. 1:**
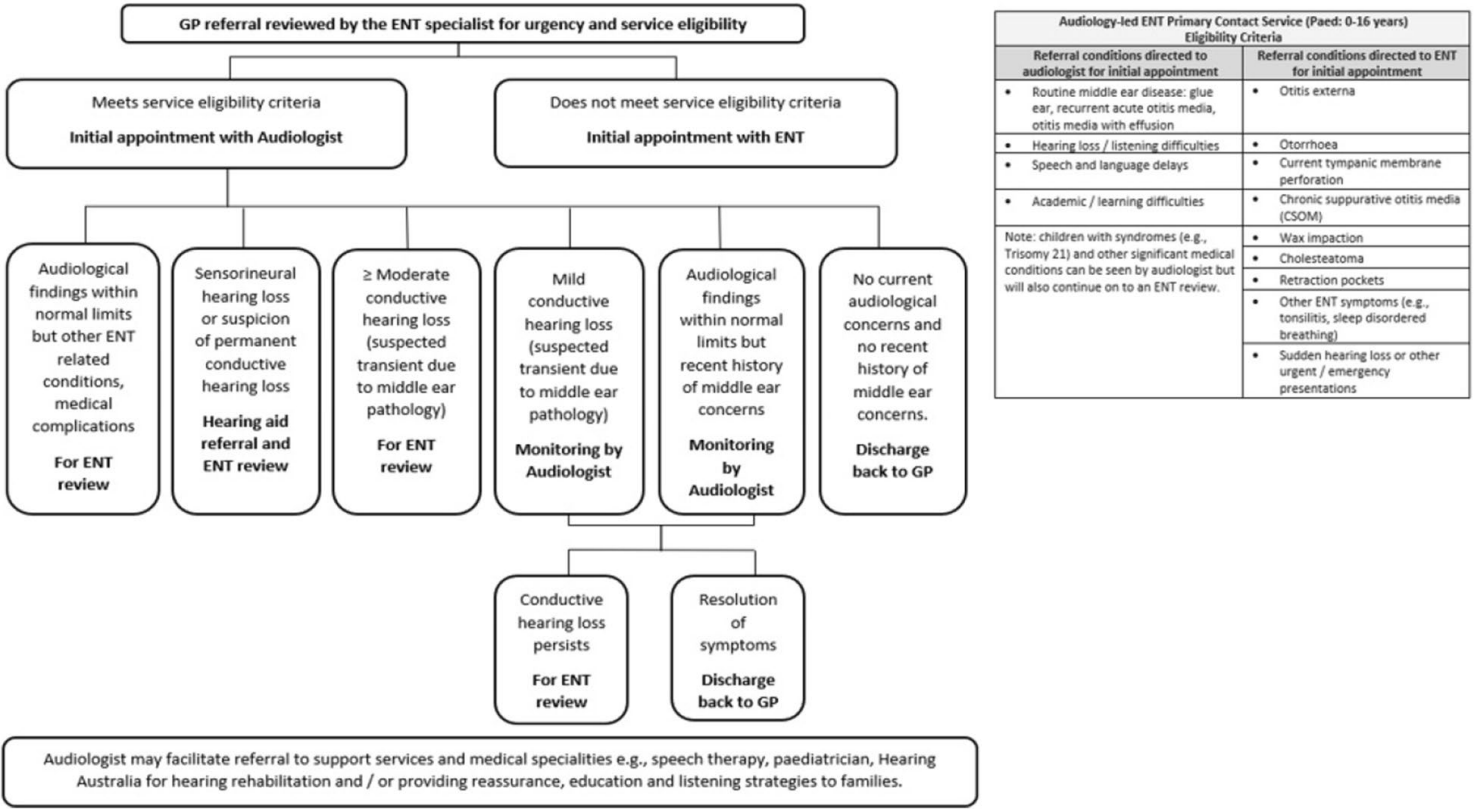
Gold Coast Health paediatric ENT audiology-led primary contact service decision pathway

All referrals into the public ENT service are sighted by an ENT specialist who determines a category of urgency and suitability for the ALP-PCS based on the referral details provided by the GP. Children aged between 0–16 years and triaged as requiring their initial appointment within 90 (category-2) or 365 days (category-3) for middle ear and hearing concerns were eligible. The ALP-PCS service eligibility criteria and decision pathway are documented in Fig. 1.

#### Study Exclusion Criteria

Patients were excluded if their original ENT referral came from another ENT specialist; if they had been assessed by an ENT specialist prior to the audiologist; if the referral did not meet service eligibility criteria due to a triaging error; or if parents withheld consent.

### Raters

The three audiologists in this study had 1 year, 3 years and 19 years clinical audiological experience at study commencement. Less experienced clinicians performed the role under the supervision of an audiologist with 19 years’ experience (principal investigator). The ENT specialist was a senior registrar with 10 years of experience in the ENT specialty.

### Clinical assessment

The ALP-PCS clinical pathway (see Fig. 1) was developed in consultation with an ENT-Allied Health steering committee and GCUH credentialling committee. Children seen in the study received audiological assessment and management in line with this agreed clinical pathway. Audiological assessment included case history, otoscopy, tympanometry and audiometry with otoacoustic emission testing performed where appropriate. Children with incomplete assessments (e.g., tympanometry not completed due to otorrhea or non-compliance) were not excluded to maintain the real-life context of the management decisions.

### Outcome measures

Following the patient’s initial audiological assessment, the audiologist completed a data collection form (see Additional file 1) and selected up to three differential site-of-lesion diagnoses from the following: outer ear pathology, middle ear pathology, Eustachian tube dysfunction, tympanic membrane pathology, patent grommet, sensorineural hearing loss, retrocochlear pathology or normal (no pathology). Adhering to the existing standard scope of practice for Australian audiologists, diagnoses were limited to a site-of-lesion rather than labelling a specific ear condition such as otitis media. The audiologists were able to select more than one diagnosis to allow for conditions that could not be differentiated without further investigation and coexisting conditions. The audiologist also selected as many management recommendations as they felt appropriate from the following; additional diagnostic audiology (such as electrophysiological testing) or repeat behavioural audiology to confirm results, audiological monitoring, ENT review, advice on water precautions, hearing aid referral (including ENT review for medical clearance), speech pathology referral, listening strategies or discharge back to the GP without ENT review.

The ENT specialist independently completed the same data collection form. As the study was conducted within bounds of the existing clinical pathway, the ENT specialist did not see the patient themselves but instead selected a up to three differential site-of-lesion diagnosis and management decisions after reviewing the relevant clinical information including: patient age and gender, referral, case history, objective audiological results and a descriptive summary of the severity and nature of the hearing loss. Raters were blinded to each other’s decisions until after data collection was finalised. Once data collection was completed, disagreements were discussed, and a joint decision agreed to ensure safe and comprehensive clinical management of study participants.

### Data analysis

Data was analysed using Stata (Version 15.1) with support from a biostatistician to identify the most appropriate coefficient for assessing level of agreement (inter-rater reliability). Percentage agreement is presented but does not take into consideration agreement by chance. Cohen’s Kappa coefficient corrects for chance agreement and is the most used measure of agreement between categorical outcomes. Cohen’s Kappa is, however, affected by a high (or low) prevalence of the trait of interest or by the existence of bias between raters in the proportion of positive ratings given [16, 17]. If either of these condition exists, alternative measures can be used such as Gwet’s first order agreement co-efficient (AC1). Gwet’s AC1 has been found to be less effected by prevalence and marginal probability than Cohen’s Kappa and is recommended for use in inter-rater reliability analysis [17].

For diagnostic agreement, we considered whether the professionals agreed on at least one differential site-of-lesion diagnosis. For recommendation agreement, we first considered whether the professionals agreed completely on the primary management decision that guided the child along the clinical pathway. The primary management decisions were; audiological monitoring, ENT review, hearing aid referral (including ENT review for medical clearance) or discharge back to the GP without ENT review. In a secondary analysis, a weighted matrix was applied to the calculation of the agreement statistic to reflect the degree of disagreement in the management decision. Recommendations for ENT review, hearing aid referral (including ENT review for medical clearance) and audiological monitoring were considered less serious disagreements in that the child was retained under the care of the hospital in all three management plans and given a weighting of 0.5 rather than 0 (indicating total disagreement). The remaining recommendations were not analysed for this paper as, on reflection by the authors, they were felt to generally preference the traditional role of the audiologist and did not impact the child’s movement along the clinical pathway in Fig. 1.

The Landis and Koch (1977) established scale was used to interpret the magnitude of percentage agreement and Gwet’s AC1 values: < 0.2 poor, 0.21–0.41 fair, 0.41–0.6 moderate, 0.61–0.8 substantial, 0.81–1 near perfect [14]. The frequency and nature of disagreements was also examined as was the patient’s outcome when a management disagreement occurred.

## Results

Fifty children with a mean age of 4.29 years (range 0.85–15.48) and 56% male (28/50) were included. Table 1 shows agreement on i) individual site-of-lesion diagnosis, ii) categorised site-of-lesion diagnosis, iii) the patient’s primary management decision, and iv) retention within the clinical pathway versus discharge back to referrer without ENT intervention.

**Table 1:** Agreement on site-of-lesion diagnosis, primary management decision and retention of patient in the primary contact clinic (*N* = 50)

	**% Agreement**		**Gwet’s AC1**		**Cohen’s Kappa**	
	**%**	**95% CI**	**Value**	**95% CI**	**Value**	**95% CI**
**Individual site-of-lesion diagnosis**	100	100–100	1.0	1.00–1.00	1.0	1.00–1.00
**Categorised site-of-lesion diagnosis**	100	100–100	1.0	1.00–1.00	1.0	1.00–1.00
**Primary management decision**	74	61.41–86.59	0.67	0.52–0.83	0.57	0.36–0.78
**Retention vs discharge**	83	74.10–91.90	0.74	0.60–0.88	0.57	0.34–0.79

**Fig. 2:**
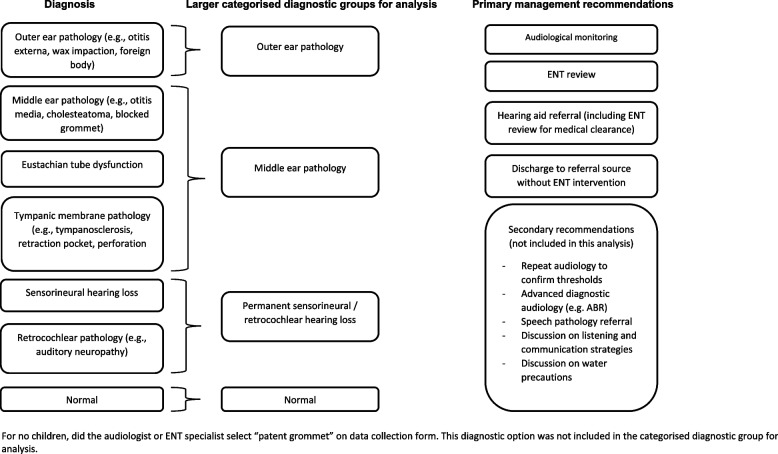
Diagnosis and management recommendation options

There was 100% agreement between the audiologist and ENT on at least one differential diagnosis in all 50 cases. In 33/50 cases, the audiologist selected only one diagnosis. The ENT was more inclined to select multiple differential diagnoses; in 32/50 cases selecting both Eustachian tube dysfunction and middle ear pathology when a middle ear site-of-lesion or conductive pathology was suspected. Considering the inconsistency in the way the rater’s approached the selection of up to three differential diagnosis, the individual differential site-of-lesion diagnoses were grouped into larger categorised diagnostic groups: outer ear pathology, middle ear pathology, permanent sensorineural/ retrocochlear hearing loss or normal (Fig. 2).

These categorised diagnostic groups better reflected the broader definitions that guide best practice management. Again, the audiologists and ENT agreed on a least one categorised diagnosis in all 50 cases. For 42 of these cases, only one categorised diagnosis was selected by each clinician. Table 2 shows concordance patterns for each of these categorised diagnoses.

**Table 2:** Differential diagnosis confusion matrix (up to 3 diagnoses able to be selected)

	Audiologists			
ENT	Differential diagnosis 1: Outer ear pathology			
		Yes	No	Total
	Yes	0	0	0
	No	0	50	50
	Total	0	50	50
	Differential diagnosis 2: Middle ear pathology			
		Yes	No	Total
	Yes	39	0	39
	No	2	9	11
	Total	41	9	50
	Differential diagnosis 3: Permanent sensorineural/retrocochlear hearing loss			
		Yes	No	Total
	Yes	1	0	1
	No	1	48	49
	Total	2	48	50
	Differential diagnosis 4: Normal (no current pathology)			
		Yes	No	Total
	Yes	11	1	12
	No	3	35	28
	Total	14	36	50

For each disagreement in the ‘normal’ diagnosis, the professionals had both nominated a concurrent ‘middle ear pathology’ differential diagnosis

Greater variability was found for the patient’s primary management decision with 74% agreement (0.67 Gwet’s AC1) between the audiologists and ENT specialist. This improved to 83% agreement (0.74 Gwet’s AC1) when the weighting was applied to reflect retention in the clinical pathway versus discharge without intervention. Based on the Landis and Koch scale, both Gwet’s AC1 calculations showed substantial agreement between the professionals on the management decision.

Table 3 illustrates the number and trend of disagreements on patient’s management within the pathway. In 9/50 cases, there was disagreement between the audiologist and ENT specialist over whether the patient should undergo further audiological monitoring or proceed directly to an ENT review, with little observable difference in the tendency of each professional to prioritise one management decision over another. Two thirds of these patients continued to surgical management after their first ENT appointment. There were no cases in this sample where the ENT specialist felt the patient required medical ENT opinion while the audiologist recommended discharge. There were four patients for whom the professionals felt there was no current pathology but disagreed about the management. In three of these four cases, the audiologist conservatively recommended further audiological monitoring whilst the ENT recommended discharge. None of these four children went on to have surgical management or be re-referred after discharge.

**Table 3:** Confusion matrix describing agreement and disagreement on primary management decision (*N* = 50)

		**Audiologist Management Decision**			
		**ENT review**	**Hearing aid referral**	**Audiological monitoring**	**Discharge**
**ENT Management Decision**	**ENT review**	16		4^a^	
	**Hearing aid referral**		1		
	**Audiological monitoring**	5^a^		17	1^b^
	**Discharge**			3^b^	3

^a^9/50 patients with pathology where audiologist and ENT specialist disagreed on whether to monitor in audiology or proceed to ENT review

^b^4 patients with no current pathology where disagreement about whether to discharge or monitor

## Discussion

Innovative AHP primary contact models of care are increasingly being utilised in public health systems to reduce long medical specialist wait times for lower acuity patients and improve patient access to timely and appropriate care [5, 10, 12, 18–20]. These models utilise experienced AHPs operating at full or advanced scope of practice to undertake specific tasks usually performed by a medical specialist.

For children with middle ear and hearing concerns, audiologists routinely work alongside ENTs and are ideally situated to provide an initial point of contact in ENT AHP models. In addition to identifying hearing loss and determining site-of-lesion for ear conditions, audiologists working at full scope provide education, counselling and reassurance alongside rehabilitation and onward referral for interventions such as speech therapy or paediatrician assessment. They also play a role in recognising risk factors for conditions that require more urgent medical management or ongoing monitoring [21].

Success of AHP primary contact models of care is enabled by a strong positive relationship between the AHPs and medical consultants, with low levels of support from medical stakeholders being a barrier to the flow of referrals into the clinics [6]. While role-substitution pathways such as the ALP-PCS are expected to have an important role in providing value-based care and meeting growing service demands, there is no agreed approach to measuring safety and quality [22]. Only a small number of studies into audiology-led ENT models have reported on safety. Pokorny et al. (2020) examined the safety of a paediatric audiology-led clinic by reporting on rerefer rates and adverse events for children discharged independently by an audiologist from an ENT specialist cohort and concluded that the risk of misdiagnosis or inappropriate discharge was minimal [10]. Zapala et al. (2010) conducted a retrospective chart review study comparing assessment and treatment plans for audiologists and ENTs in older adult patients and concluded that there was no convincing evidence that audiologists missed significant symptoms of otologic disease and did refer to ENTs when appropriate [23]. In this study, we used a measure of concordance between the audiologist and ENT on site-of-lesion diagnosis and management to examine decision making competence. This methodology is comparable to publications from musculoskeletal services showing high level of agreement between medical specialists and physiotherapists working in primary contact models [18, 20, 24, 25]. As with those studies, we also found substantial agreement between audiologists and the ENT specialist regarding onward management of children with middle ear or hearing concerns. This agreement was near perfect for site-of-lesion diagnosis. The risk of misdiagnosis and inappropriate management was minimal, and no children were independently discharged from care by the audiologist when the ENT felt medical opinion was warranted.

Previous research has shown that the ALP-PCS pathway significantly reduces the time children with middle ear concerns wait for their first appointment, thus improving timely access to care [5]. The shorter waiting time allows for an additional point of triage to identify higher acuity conditions, such as undiagnosed permanent childhood hearing loss or sudden hearing deterioration, which can be missed in initial referral triage due to insufficient referral information. For children with chronic pathology, thorough audiological work-up prior to the ENT review informs candidacy for surgical intervention [26]. We found that the audiologist and ENT specialist agreed on at least one categorised diagnosis for all children (Table 2), which guided the child’s progress along an evidence-based decision pathway for appropriate clinical care (Fig. 1). Previous research which examined diagnostic agreement for otitis media between experienced audiologists and ENT specialists showed similar high levels of diagnostic agreement [27]. It is likely that interpretation of the audiogram and other objective audiological testing supports audiologists in correctly identifying the site-of-lesion and nature of the underlying pathology. When developing these services, other measurable criteria can be agreed upon by the audiologist and ENT, such as the number of ear infections or degree of hearing loss, to guide the audiologist around the urgency of medical opinion, leading to earlier escalation of care for children suspected of more chronic or complex conditions.

Our study found substantial agreement (83%; 0.74 Gwet’s AC1) on the onward management of patients when deciding between whether a child needed to stay within the clinical pathway or could be discharged back to the referral source without medical opinion. However, in a subset of discordant cases (9/50) there was variability in decisions for children that stayed within the clinical pathway around the need for immediate medical review to consider surgical intervention or whether a watchful waiting period with audiological monitoring was more appropriate. Neither professional demonstrated a clear bias towards conservative monitoring or medical review. Whilst a watchful waiting period is endorsed as best practice to ensure that surgical management of otitis media is reserved for persistent or recurrent cases, in practice, the medical management of otitis media varies and decisions to intervene should take into consideration the context of the child, including factors such as age, risk of complications, functional hearing difficulties, and parental preference [28, 29]. An acknowledged limitation of the study was that the ENT did not assess the patient in person, leaving the ENT to make management decisions based on written information only. It is likely that this would have hindered the ENT in exploring some of the more subjective factors that influence recommendations or perhaps led to overcaution on the behalf of the ENT in forming a diagnosis. Certainly, this may explain the tendency in this study for the ENT specialist to select more differential diagnosis than the audiologist. Alternatively, audiologists and ENTs bring different perspectives to the appointment and variability may have come from the profession of the rater. Whilst the audiologist is an advocate for timely management of persistent middle ear conditions to avoid the associated implications of ongoing hearing loss, the ENT is best placed to consider factors such as age, risk of complications, surgical readiness, coexisting ENT conditions such as recurrent tonsillitis and prioritisation of middle ear conditions amongst other pressing ENT cases such as obstructive sleep apnoea or head and neck cancer.

There were four asymptomatic children at the time of the initial consultation where the audiologist was more likely than the ENT to recommend conservative monitoring over discharge. This is likely due to the requirement in the ALP-PCS decision pathway to monitor children with a history of recent middle ear issues before discharge and represented a safe, conservative approach by the audiologist (Fig. 1). Previous research has found that experienced audiologists have the confidence to discharge children seen in an Audiology-led primary contact model without a second medical opinion [10]. By discharging children without current pathology back to their GP for conservative monitoring, an unnecessary review appointment which would otherwise negate some of the demonstrated cost effectiveness benefits of primary contact services [5, 30] can be avoided. It is proposed that the development of an accelerated re-referral pathway should symptoms recur in currently asymptomatic children may increase the confidence of audiologists to discharge at the initial appointment, improve recommendation consistency between the professionals and streamline patient care. Further investigation into re-referral rates, both in the current service and if an accelerated re-referral pathway was implemented, is recommended to provide additional evidence to the overall safety of an ALP-PCS.

To our knowledge, this is the first published study examining interprofessional agreement between audiologists and ENT specialists in the diagnosis and management of patients within an ALP-PCS. Our findings indicated that the ENT made comparable diagnostic and management decisions to the audiologist when the ENT was provided the chance to review the patient’s relevant clinical information. Whilst there are acknowledged limitations in the design of this study, including small sample size, absence of video-otoscopy, limited number of raters and dependence on clinical notes only for ENT decision making, these preliminary findings offer reassurance about the competency of audiologists to accurately identify hearing and middle ear issues upon which the clinician can base safe onward management decisions. Further research on interrater reliability utilising a design that includes children being independently assessed, in person, by a broader group of ENT specialists and audiologists is encouraged, as well as the benefits of ALP-PCS including patient re-referral rates, economic analysis, patient reported outcomes and experience measures, and impacts on ENT service activity in patients with higher complexity would provide further knowledge about these innovative models of care. Research into the application of ALP-PCS beyond the public hospital setting may also inform workforce alternatives in bringing hearing health services to regional and remote areas where, in Australia, high rates of childhood otitis media is an ongoing challenge for sparce ENT services [31]. Certainly, the improved access and reduced waiting times associated with these services provides children with faster access to hearing assessment and management supporting speech, language and developmental outcomes; and this exploratory study is a further step in building confidence in the efficacy and safety of an alternative audiology-led clinical pathway for paediatric ENT patients with middle ear and hearing concerns.

## Conclusions

Audiology-led Paediatric Primary Contact services utilise collaborative practice between Audiologists and ENTs though a role substitution model to help manage lengthy ENT waiting lists. Our research provides further evidence that this novel approach for children with middle ear and hearing concerns is safe as well as efficient.

## Supplementary Information

**Additional file 1.** Data collection form.

## Data Availability

The datasets analysed during the current study are available from the corresponding author on reasonable request.

## Abbreviations

ENT: Ear, Nose and Throat
GCUH: Gold Coast University Hospital
AHP: Allied Health Practitioner
ALP-PCS: Audiology-led Paediatric Primary Contact Service
GP: General Practitioner
