# Supplementary figures and images for: Evaluating diagnostic and management agreement between audiology and ENT: a prospective inter-rater agreement study in a paediatric primary contact clinic
Source: BMC Pediatr. 2022 Nov 8;22:646. doi: 10.1186/s12887-022-03695-3 (PMC9641870; doi:10.1186/s12887-022-03695-3)

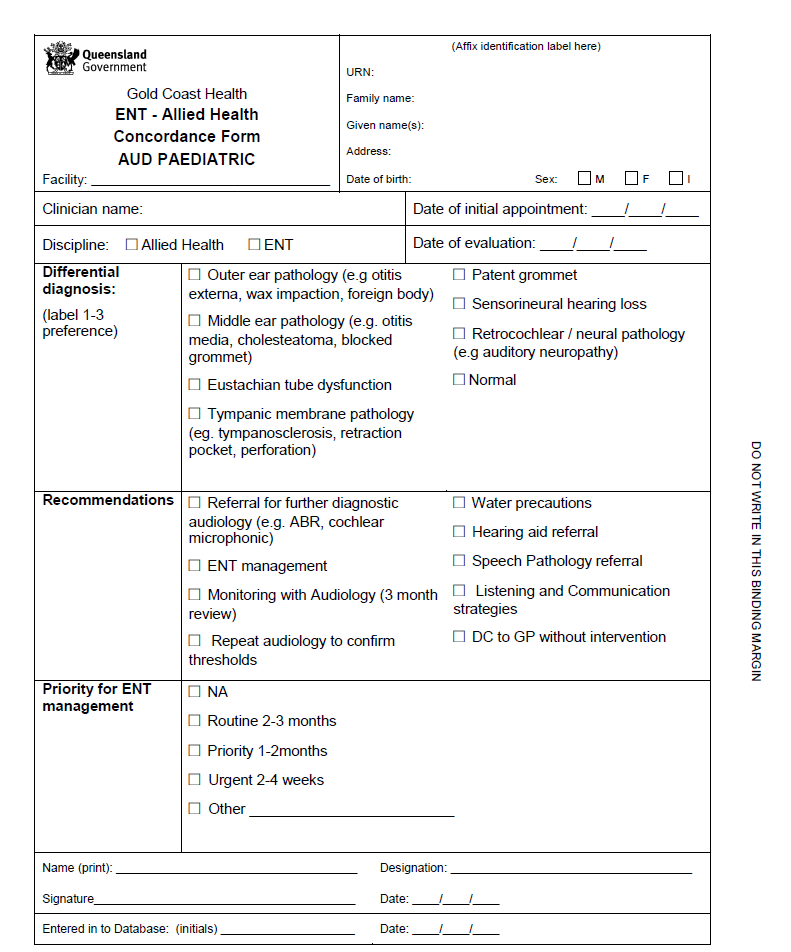
**Additional file 1. Data collection form**

Supplement: Supplementary file 1 — Additional file 1. Data collection form. [file 12887_2022_3695_MOESM1_ESM.docx]
